# An international trial of quantitative PCR for monitoring *Legionella* in artificial water systems

**DOI:** 10.1111/j.1365-2672.2011.04957.x

**Published:** 2011-04

**Authors:** JV Lee, S Lai, M Exner, J Lenz, V Gaia, S Casati, P Hartemann, C Lück, B Pangon, ML Ricci, M Scaturro, S Fontana, M Sabria, I Sánchez, S Assaf, S Surman-Lee

**Affiliations:** 1 Health Protection AgencyLondon, UK; 2 Institute for Hygiene and Public Health, Universität BonnBonn, Germany; 3 Istituto Cantonale di MicrobiologiaBellinzona, Switzerland; 4 CHU NancyNancy, France; 5 Institute of Medical Microbiology and Hygiene, University of TechnologyDresden, Germany; 6 Unité de Microbiologie-Hygiène, CH VersaillesVersailles, France; 7 Istituto Superiore di SanitàRoma, Italy; 8 Autonomous University of BarcelonaBarcelona, Spain; 9 Pall GeneSystemsBruz, France

**Keywords:** international trial, interpretation, *Legionella*, monitoring, qPCR, water

## Abstract

**Aims:**

To perform an international trial to derive alert and action levels for the use of quantitative PCR (qPCR) in the monitoring of *Legionella* to determine the effectiveness of control measures against legionellae.

**Methods and Results:**

Laboratories (7) participated from six countries. Legionellae were determined by culture and qPCR methods with comparable detection limits. Systems were monitored over ≥10 weeks. For cooling towers (232 samples), there was a significant difference between the log mean difference between qPCR (GU l^−1^) and culture (CFU l^−1^) for *Legionella pneumophila* (0·71) and for *Legionella* spp. (2·03). In hot and cold water (506 samples), the differences were less, 0·62 for *Leg. pneumophila* and 1·05 for *Legionella* spp. Results for individual systems depended on the nature of the system and its treatment. In cooling towers, *Legionella* spp. GU l^−1^ always exceeded CFU l^−1^, and usually *Legionella* spp. were detected by qPCR when absent by culture. The pattern of results by qPCR for *Leg. pneumophila* followed the culture trend. In hot and cold water, culture and qPCR gave similar results, particularly for *Leg. pneumophila.* There were some marked exceptions with temperatures ≥50°C, or in the presence of supplementary biocides. Action and alert levels for qPCR were derived that gave results comparable to the application of the European Guidelines based on culture. Algorithms are proposed for the use of qPCR for routine monitoring.

**Conclusions:**

Action and alert levels for qPCR can be adjusted to ensure public health is protected with the benefit that remedial actions can be validated earlier with only a small increase in the frequency of action being required.

**Significance and Impact of the Study:**

This study confirms it is possible to derive guidelines on the use of qPCR for monitoring the control of legionellae with consequent improvement to response and public health protection.

## Introduction

Legionellosis is a group of infections caused by bacteria of the genus *Legionella.* The most severe is Legionnaires’ disease, an acute pneumonia that often leads to death and was first recognized in 1976 (Fraser *et al.* 1977). Outbreaks and sporadic infections occur throughout the world. Between 2005 and 2006, there were 11 980 cases reported from 35 countries in Europe (Ricketts and Joseph 2007). At least 50 species of *Legionella* have been described, and 20 have been associated with disease in man, but by far the most common cause of Legionnaires’ disease is *Leg. pneumophila* (Bartram *et al.* 2007). *Legionella* species are opportunistic pathogens of humans which normally inhabit warm moist or aquatic environments where they grow in association with other organisms. In particular, they are known to grow in a range of protozoa. Their predilection for warm water means that they are capable of colonizing artificial water systems and equipment containing water. Legionnaires’ disease is not transmitted from person to person but is of environmental origin and usually contracted by inhaling the organism in an aerosol produced from water contaminated with the organisms or aspiration of contaminated water particularly in hospitals. The most common sources of infection are cooling towers and evaporative condensers, hot and cold water systems and spa pools, but a variety of other artificial sources have also been described (Bartram *et al.* 2007).

The environmental origin of Legionnaires’ disease was identified soon after the description of the disease. It is now recognized that infections can be prevented by the appropriate design, construction and maintenance of water systems, and other equipment using water, so as to minimize the opportunities for legionellae to grow in them and be released from them. In some countries, there is a legal requirement to take specific measures to prevent Legionnaires’ disease for example in the UK (Anon 2000), and guidelines for the prevention of Legionnaires’ disease associated with travel have been produced and adopted by most countries in Europe (Joseph *et al.* 2005). Nowadays, particularly in Europe, sampling for *Legionella* species is widely undertaken to monitor the effectiveness of control measures and sometimes for regulatory purposes. Currently where national regulations or guidelines exist, these include a quantitative measurement based on culture by the international standard ISO 11731 (Anon 1998) or a similar national standard. The culture method is complex involving concentration of micro-organisms from water by filtration and/or centrifugation followed by heat and acid pretreatments and culture on a selective medium GVPC [buffered charcoal yeast extract agar (BCYE) with selective supplements glycine, Vancomycin, Polymixin and Cycloheximide]. It can take up to 14 days to obtain a confirmed result by culture, and the results are often variable with poor recovery.

Quantitative polymerase chain reaction (qPCR) has been developed for real-time monitoring of *Legionella* in water systems and is both rapid, specific and sensitive. The qPCR methods can be applied to both the routine monitoring of water supply systems and for the follow-up of disinfection treatments (Anon 2010, 1998, Alleron *et al.* 2008; Dusserre *et al.* 2008, Joly *et al.* 2006). However, the interpretation of qPCR results has been largely controversial (Yamamoto *et al.* 1996). Several studies comparing culture and real-time PCR methods in different water types showed a higher rate of positive results and higher quantification values with real-time PCR compared to the standard culture method (Behets *et al.* 2007; Buchbinder *et al.* 2002; Joly *et al.* 2006; Levi *et al.* 2003; Wellinghausen *et al.* 2001; Yamamoto *et al.* 1993, 1996; Yaradou *et al.* 2007). There are several reasons postulated for this apparent difference including the detection of postdisinfection, sub lethally damaged cells, which are still viable but not culturable (Alleron *et al.* 2008; Dusserre *et al.* 2008). Shih and Lin 2006 suggested that remaining nucleic acids in dead cells might still be recovered and amplified by PCR. An alternative explanation for the difference between qPCR and culture for the detection of legionellae may be that culture is optimized to detect *Leg. pneumophila* serogroup 1 and does not detect all the *Legionella* species present in a system. This may be particularly relevant for samples taken from cooler systems or parts of systems (operating at <37°C) such as from cooling tower ponds.

The Association Française de Normalisation (AFNOR) has developed a standard, NF T90-471, to help ensure the equivalence of results obtained by different qPCR assays (Anon 2010). This is being further developed as a new international standard by the International Organization for Standardization (ISO). Some commercially available assays have been certified to NF T90-471. With such assays, it should be possible to obtain a result within a few hours of sampling with associated benefits to water management and public health.

A major problem exists in using these assays for compliance testing in that the action levels for positive *Legionella* counts in national legislations and the European and WHO Guidelines are based on culture (Anon 2000, Joseph *et al.* 2005; Bartram *et al.* 2007). There is currently no consensus on how qPCR results should be translated into these culture-based limits or otherwise interpreted. If a standard qPCR protocol is considered to be a good tool for monitoring *Legionella* in water systems, there must be agreement on how the results are interpreted. The objective of this study was to carry out an international multicentre trial to define the action thresholds of real-time PCR for the monitoring of legionellae in different types of water systems and thereby to facilitate interpretation of environmental legionella monitoring results using the latest standardized qPCR methods.

## Materials and methods

### Participating laboratories and selection of sample sites

Seven laboratories from six countries (France, Germany, Italy, Spain, Switzerland and the United Kingdom) participated in the study. Each laboratory was requested to sample at least six systems, weekly for a minimum of 6 weeks. The samples collected included some examples of the water supplied to the system as well as samples representative of the system itself. Systems were selected that were expected to give some positive results because they were known to have been colonized with legionellae previously. Each laboratory was requested to examine both cooling tower systems and domestic hot and cold water systems, if possible at least three of each. In addition, some laboratories also examined samples from spa pools and hot tubs.

Water samples of 2000 ml from hot and cold water systems or 500 ml from cooling systems were collected in accordance with ISO 19458:2006 into sterile containers containing sodium thiosulphate to neutralize any residual oxidizing biocides in the water. Samples were transported to the laboratory as soon as possible and processed within 24 h of collection.

### Sample analysis

Each sample was mixed well by shaking by hand then divided into two equal portions. One portion was assayed by qPCR for *Legionella* spp. and *Leg. pneumophila* using the *Legionella* method of Pall GeneSystems, Bruz, France which is certified by AFNOR as complying to the French standard NF T90-471.

Briefly, bacterial DNA from 250 ml of cooling tower water (CTW) and 1000 ml of hot or cold water from domestic water systems (DW) was purified using the GeneExtract instrument, which can process 47 water samples and one negative control simultaneously (Pall Genesystems). qPCR was performed with the GeneDisc® Cycler with the *Legionella* DUO GeneDisc® plate, which incorporates six analytical sectors for the analysis of five DNA extracts from water samples and one negative control. Each sector of the plate incorporates six analytical sectors for the analysis of five DNA extracts from water samples and one negative control of the entire method. Each sector consists of six PCR wells preloaded with specific primers and probes according to AFNOR NF-T90-471. Briefly, each of the five sectors dedicated to the sample analysis allows *Leg. pneumophila* and *Legionella* spp. quantification in duplicate and includes an internal inhibition control for *Leg. pneumophila*, an internal inhibition control for *Legionella* spp. and a negative PCR control. The sixth sector is dedicated to the negative control of the entire method and also includes external quantitative controls of *Leg. pneumophila* and *Legionella* spp., respectively.

The second portion was assayed by culture for *Legionella* species following ISO 11731. The flora from the water sample was concentrated by filtration or centrifugation and resuspended in 10-ml sterile water or Page’s saline and 0·270-ml portions of concentrate cultured onto the selective medium GVPC without pretreatments and after acid or heat pretreatment. The volume of concentrate used to inoculate the selective medium (0·270 ml) was selected so that equivalent volumes of the unconcentrated original water sample were examined by each method (27 ml for water from hot and cold water systems and 6·7 ml for water from cooling towers).

### Ring trial

The participating laboratories were experienced in the detection and isolation of legionellae by culture and demonstrated competence by their performance in external quality assurance schemes for legionella isolation. To ensure that all laboratories were able to use the qPCR methods reliably, a ring trial was performed at the beginning of the study. Two DNA samples corresponding to 10^2^ and 10^3^ genome units (GU) of *Leg. pneumophila* sg1 ATCC33152 per PCR well and two water samples from a hot water system spiked with 2 × 10^4^ and 2 × 10^5^ CFU l^−1^ of *Leg. pneumophila* sg1 ATCC 33152 were distributed to all participants to be analysed in duplicate, on the same day.

For each laboratory, the average bias (*b*), the standard deviation of repeatability (*S*_r_) and the uncertainty *U* [
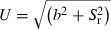
] were calculated. For the data set, including each laboratory data, the average bias, the standard deviation of reproducibility (*S*_R_) and the uncertainty were calculated.

### Analysis of results

For each sample, purified DNA was eluted in a final volume of 150 μl of elution buffer. Each analytical well was filled with a mixture of DNA extract (6 μl) and Master mix solution (6 μl) provided with the kit. The limit of detection (LOD) was 5 GU per well corresponding to 750 GU l^−1^ for samples from cooling towers and 190 GU l^−1^ for samples from hot and cold systems. The limit of quantification was 25 GU per well corresponding to 3750 GU l^−1^ for samples from cooling towers and 940 GU l^−1^ for hot and cold water samples. For culture, the detection limit was taken to be 750 CFU l^−1^ for cooling towers and 190 CFU l^−1^ for hot and cold water samples. The presence of five colonies detected on the growth medium in the aliquot specified ensures there is a >90% probability of subsequent aliquots from the same suspension yielding at least one colony.

All results were entered on a standardized data base and analysed using Microsoft Excel. The positive and negative predictive values (PPV, NPV) of the qPCR technique were calculated. PPV corresponds to the ratio between the number of culture-positive samples and the number of positive samples by both methods. NPV corresponds to the ration between culture-negative samples and the total number of negative samples. The comparison of the quantitative results was made on the samples that were positive with both culture and qPCR methods. These values were then plotted as a distribution of the logarithmic difference between the results obtained by qPCR and those obtained by culture.

## Results

### Ring trial

All the laboratories performed well, and the standard deviation was <0·2 for both the two DNA samples and the two water samples This was below the standard deviation of 0·25 recorded during the AFNOR validation of the GeneDisc *Legionella* method. The global uncertainties were 0·24 and 0·33 for the DNA samples and 0·36–0·37 for *Leg. pneumophila* and 0·38–0·41 for *Legionella* species in the water samples. It was concluded that the qPCR was performing satisfactorily and comparably in each laboratory.

### Overall comparison of qPCR results with culture

**Figure 1:**
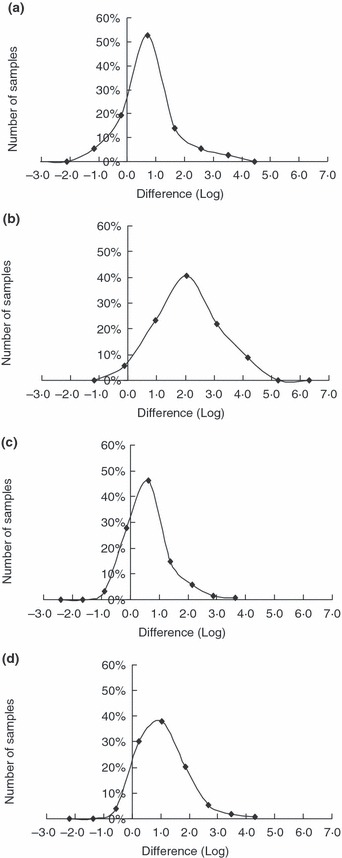
Distribution of logarithmic differences between quantitative PCR result (GU l^−1^) and culture results (CFU l^−1^) in water samples (a) *Legionella pneumophila* in cooling towers –*Leg. pneumophila*– detected by both methods in 36/232 pairs of samples (b) *Legionella* species in cooling towers –*Legionella* species detected by both methods in 69/232 pairs of samples (c) *Leg. pneumophila* in domestic hot and cold water systems –*Leg. pneumophila* detected by both methods in 154/506 pairs of samples. (d) *Legionella* species in domestic hot and cold water system samples –*Legionella* species detected by both methods in 239/506 pairs of samples.

Combining the results from all of the laboratories, there were 232 pairs of results for samples from cooling towers and 506 pairs of results for samples from hot and cold water systems. PCR inhibitors were overcome by carrying out a tenfold dilution. However, where this was necessary, this dilution increased the LOD by qPCR causing a lack of comparability in sample volumes examined by PCR and culture for these specimens. Consequently, results for 20 samples from DWs and eight samples from cooling towers were excluded from the analysis. In Fig. 1, only pairs of results with readings above the quantification limit have been used. The amount of *Legionella* DNA determined as GU l^−1^ by qPCR was generally higher than the concentration of legionellae estimated as CFU l^−1^ using culture (Fig. 1). The difference was greatest for *Legionella* spp. in CTWs (Fig. 1b) for which the mean log difference was 2·03 (SD 1·07) based on 69 pairs of samples in which *Legionella* spp. were detected by both methods. In contrast for *Leg. pneumophila* in cooling tower samples (Fig. 1a), there were 36 pairs of results for which the mean log difference was 0·71 (SD 0·94). In samples from domestic hot and cold water systems, the mean log difference was 0·62 (SD 0·76) for *Leg. pneumophila* based on 154 pairs of results and 1·05 (SD = 0·81) for *Legionella* spp. based on 239 pairs of results (Fig. 1c,d).

The ability of the qPCR results to predict the presence or absence of *Legionella* spp. and *Leg. pneumophila* as detected by culture results was also investigated. For this analysis, any result above the detection limit was considered positive. The results are summarized in Table 1, and these were used to calculate the PPV, NPV, sensitivity and specificity of qPCR as a means of predicting the culture results and are shown in Table 2.

**Table 1:** Comparison of results for the presence or absence of *Legionella* or *Legionella pneumophila* as determined by quantitative PCR (qPCR) and culture

	Results by culture			
	
	*Legionella* spp.		*Leg. pneumophila*	
		
Results by qPCR	Detected	Not detected	Detected	Not detected
Cooling towers				
Detected	73 (31)*	148 (64)	62 (27)	52 (22)
Not detected	0 (0)	11 (5)	3 (1)	115 (50)
Hot and cold water				
Detected	278 (55)	217 (43)	249 (49)	168 (33)
Not detected	3 (1)	7 (1)	10 (2)	78 (15)

* Number (%) of samples.

**Table 2:** Ability of quantitative PCR to predict the culture result

	Cooling towers		Hot and cold water systems	
		
	*Legionella* spp. (%)	*Legionella pneumophila* (%)	*Legionella* spp. (%)	*Leg. pneumophila* (%)
Positive predictive value	33	54	56	60
Negative predictive value	100	97	70	89
Sensitivity	100	95	99	96
Specificity	7	69	3	32

### Analysis by individual source

**Figure 2:**
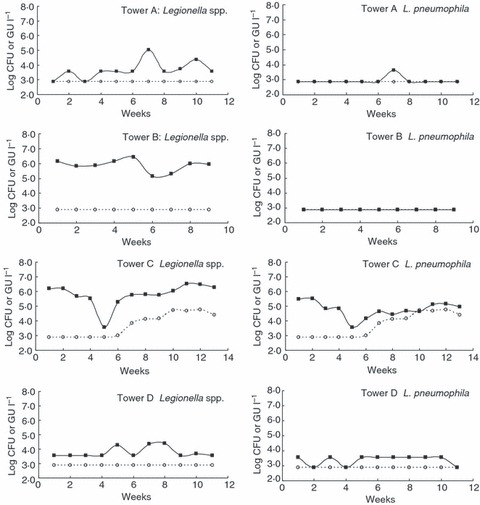
Weekly monitoring of cooling towers for *Legionella* spp. and *Legionella pneumophila* by culture (○) and quantitative PCR (▪). The log minimum detectable was 2·9 (750) CFU l^−1^ or GU l^−1^. Biocide treatments were: tower A, chlorine; tower B bromine and isothazolones; tower C, ozone and tower D bromine.

**Figure 3:**
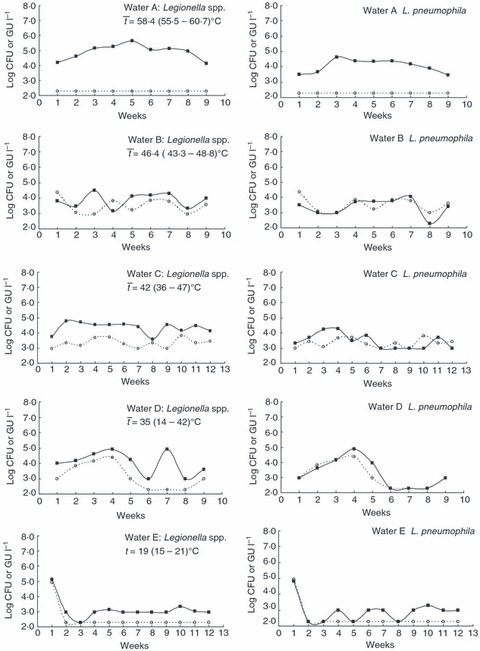
Weekly monitoring of domestic hot and cold water outlets for *Legionella* spp. and *Legionella pneumophila* by culture (○) and quantitative PCR (▪). The log minimum detectable was 2·3 (190) CFU l^−1^ or GU l^−1^. In addition to heat, water B was also treated by copper/silver ionization and water E with chlorine dioxide.

The performance of qPCR and culture for routine weekly monitoring of individual water systems over at least 10 weeks was also compared. Examples of some typical results for cooling towers are shown in Fig. 2 and for some domestic water outlets in Fig. 3. Careful review of the data indicates that the results for individual systems are dependent on the nature of the system and its treatment. The marked difference between the results for *Legionella* spp. and *Leg. pneumophila* for cooling towers is clear in Fig. 2. Generally, the GU l^−1^ for *Legionella* spp. is much greater than the corresponding CFU l^−1^ detected by culture, and usually *Legionella* spp. were detected by qPCR even when there were no legionellae detected by culture. In contrast, when a cooling tower was adequately maintained on a suitable biocide regime and *Leg. pneumophila* was not detected by culture, then qPCR also failed to detect *Leg. pneumophila* or gave a low value. This is illustrated by towers A, B and D in Fig. 2. When *Leg. pneumophila* was detected by culture, then the pattern of results for qPCR followed the same trend as can be seen for tower C in Fig. 2. However, this also illustrates another observation which was that, in the absence of detection by culture, high qPCR results often precede detection by culture.

In samples from domestic hot and cold water systems, the results for culture and qPCR were often similar, particularly for *Leg. pneumophila* (see Fig. 3). However, there were some marked exceptions such as water A in Fig. 3. A close examination of the data indicated the greatest discrepancies between results from qPCR and culture occurred more frequently in samples taken from systems with a high water temperature. Data from 275 samples from hot and cold water systems, for which there was adequate temperature data, were analysed to investigate the influence of temperature on the results. The mean log differences between the results for qPCR as GU l^−1^ and culture as CFU l^−1^ at different temperatures are shown in Table 3. At temperatures above 50°C, the mean log differences were significantly higher (*P* < 0·05) both for *Legionella* spp. and for *Leg. pneumophila* than at lower temperatures.

**Table 3:** Analysis of mean log differences between quantitative PCR (GU l^−1^) and culture (CFU l^−1^) results for water samples from building domestic water systems at different temperature ranges

		*Legionella* spp.			*Legionella pneumophila*		
			
*T*° range	Number of samples	Mean log PCR	Mean log culture	Mean log difference	Mean log PCR	Mean log culture	Mean log difference
≤25°C	20	3·66	2·51	1·15	2·77	2·37	0·40
25–30	13	3·65	2·60	1·05	2·76	2·52	0·24
30–35	24	3·88	2·77	1·11	3·11	2·71	0·39
35–40	24	4·28	2·92	1·36	3·18	2·83	0·34
40–45	45	4·34	3·07	1·28	3·20	3·01	0·19
45–50	29	4·05	3·03	1·02	3·35	3·03	0·31
50–55	30	4·47	2·64	1·83*	3·61	2·60	1·01*
55–60	69	4·42	2·52	1·90*	3·60	2·52	1·07*
≥60°C	21	4·43	2·30	2·13*	3·44	2·28	1·16*

Results were analysed for 275 samples.

* Result significantly different (*P* < 0·05 *T* test) to values for lower temperatures.

In some samples, there was a difference between qPCR and culture at low temperatures. An example is water E in Fig. 3 taken from a system which was treated continually with chlorine dioxide as a supplementary disinfectant. The first water sample collected from this monitoring point had high and equivalent levels of *Leg. pneumophila* (approximately 10^5^) by both qPCR and culture. As a consequence, the corresponding outlet was cleaned, dismantled and disinfected. *Legionella pneumophila* was subsequently not detected by culture over the next 11 weeks although qPCR was intermittently positive from the third week onwards at the levels of about 10^3^ GU l^−1^.

## Discussion

As noted in the introduction, others have observed that legionellae are detected in a higher proportion of water samples by qPCR than by standard culture methods such as ISO11731. This is particularly true when the target is the genus *Legionella* as opposed to *Leg. pneumophila*. As expected, this difference is also reflected when comparing the concentration of legionellae determined by qPCR (GU l^−1^) with the concentration determined by culture (CFU l^−1^). In this study, for samples from cooling towers, there was a significant difference between the log mean difference between the qPCR result expressed as GU l^−1^ and the culture result (CFU l^−1^) for *Leg. pneumophila* (0·71) and for *Legionella* spp. (2·03). In hot and cold water from DWs, the differences were much less, 0·62 for *Leg. pneumophila* and 1·05 for *Legionella* spp.

The detection by qPCR of apparently higher levels of legionellae is often considered to be an argument against the use of PCR for routine monitoring because of the difficulty of interpreting qPCR results against the quantitative limits based on culture contained within legislation and guidance documents. The purpose of this study was to gather information to support the development of guidelines for the interpretation of qPCR results. There are several possible explanations why one would not expect qPCR and culture to give closely equivalent values with natural samples. The population may include injured, dying or dead organisms that are no longer capable of growth on artificial media but still contain DNA and so may be detected by PCR. The culture method is complex with many steps including concentration, resuspension, pretreatment with acid or heat and inoculation onto a selective medium containing a variety of antibiotics. There may be considerable losses of legionellae during these processes. In addition, their growth can sometimes be inhibited by the presence of other organisms on the selective agar. Thus, culture does not recover all of the *Legionella* cells within a sample, and indeed, results from laboratory comparisons show that they commonly only achieve recoveries in the range 10–60% (Lee *et al.* 2002). There may also be viable but nonculturable cells in a population that are capable of multiplying in nature but not on artificial media. The genome divides before the cell so that an average of more than one gene copy per cell may be present particularly in actively growing populations. For qPCR, there may be some losses during concentration and extraction, but conformity with NF T90-471 specifies that the recovery by qPCR should be >25%. During the AFNOR validation of the *Legionella* GeneDisc, recoveries were shown to be 97% for mineral water, 99% for hot sanitary water and 84% for CTW. There is the possibility of inhibitors of the PCR being present in the sample, but these can be readily detected by appropriate internal controls, whereas there are no similar internal controls available to enable the ready detection of poor recovery by culture. It is not surprising therefore that the quantities of legionellae detected by qPCR rarely equate to those detected by culture. Furthermore, for *Legionella* spp., the situation is probably worse as the isolation methods were originally developed for the detection of *Leg. pneumophila*, the species most commonly isolated from cases of infection, and not for other species of environmental origin growing at lower temperatures. Some species do not grow or grow only weakly at 36°C, the temperature commonly used to isolate *Leg. pneumophila* and the other pathogenic species. The isolation medium is also not suitable for some of the other species that only grow poorly if at all on the *Legionella* growth medium BCYE particularly when selective agents are present as in GVPC (Lee *et al.* 2002; Bartram *et al.* 2007).

The genus *Legionella* is already very large with at least 50 species and probably more will yet to be described that may be detected by PCR but not by culture. The aquatic environment contains vast numbers of species and genera of bacteria undoubtedly including many that are yet to be detected, isolated and described. It is therefore also possible that the gene targets used to detect *Legionella* spp. may cross react with these as yet unrecognized species. Certification to NF T90-471 requires kit suppliers to demonstrate the specificity of their test by reaction with 36 strains of *Legionella* representing a variety of species and exclusivity by failing to react with 17 non-*Legionella* strains usually encountered in the same ecosystems. The validation of the GeneDisc kits used in this study exceeded these certification requirements: 74 *Legionella* strains including 29 natural strains were tested for inclusivity, and 36 non-*Legionella* strains including 10 natural (environmental) strains were tested for exclusivity.

The distribution of bacteria in water is random and would be expected to follow Poisson, but in reality, organisms tend to be overdispersed for example because of clumping and therefore the distribution is usually greater than predicted by Poisson (Cooke *et al.* 1995). The alert levels in some guidelines are equivalent to only a few colonies being detected on a culture plate. At these levels, there is considerable potential for natural variation in the number of colonies detected in different subsamples. For example, if six colonies are detected in an aliquot from a sample, there is a 95% probability that a second aliquot from the sample will yield between 1 and 16 colonies (Anon 2002). This natural variation is likely to be exaggerated by the variable and poor recovery of the culture method. For a count to be statistically valid, it is generally considered that there should be at least 10 colonies on the plate (Anon 2007). This is recognized in the French standard method in that *Legionella* counts are only reported if at least five colonies are counted. If less than five colonies are detected, then the report states ‘*Legionella* detected’ without specifying a count. These factors combined will exacerbate the apparent discrepancies between well-validated qPCR methods conforming to NF T90-471 and the less well-validated culture methods for which the limits of detection and quantification have not been clearly delineated.

In view of the apparent lack of correlation between qPCR and culture, it is important to analyse data from the routine monitoring of water systems to establish whether it is possible to derive action and alert levels for qPCR results that, in practice, will achieve overall comparability with culture in terms of the actual actions required to be taken by maintenance engineers and water treatment specialists in response to adverse testing results.

We examined our data to see whether it is possible to derive action and alert levels for qPCR that overall yield results that are comparable to those derived from the application of the commonly used culture action and alert levels. In the European Guidelines (Joseph *et al.* 2005), it is stated that, for cooling towers, if the count of legionellae is above 10 000 CFU l^−1^, the system should immediately be re-sampled and ‘shot dosed’ with an appropriate biocide and the risk assessment and control measures be reviewed to identify appropriate remedial actions. At levels above 1000 CFU l^−1^, resampling is recommended and if the result is repeated, the control measures should be reviewed to identify whether additional control measures are required. In Table 4, the alert and action levels for qPCR were selected to allow for the overall mean difference in results for qPCR and culture. In adjusting the qPCR actions levels, the target was to achieve as high a proportion of results in the boxes indicating agreement in the actions required and to minimize any results in those corresponding to complete disagreement, i.e. when culture indicates no action is required and qPCR indicates emergency immediate action or *vice versa*. In practice, the PCR targets selected were the levels used for culture adjusted by the corresponding mean difference found in this study, i.e. for *Leg. pneumophila* in cooling towers the alert level was taken to be 5× greater (equivalent to a log difference of 0·71) than the corresponding culture targets and in hot and cold water 4× greater (equivalent to a log difference of 0·61). Using the *Leg. pneumophila* target, for 77% of comparisons, the use of the two tests would have resulted in identical responses. For 20% of comparisons, there was partial disagreement with culture more commonly (11%) indicating an alert response when qPCR was satisfactory as opposed to 4% of samples when the reverse was true. An alert response would normally be a retest and review of the management of the system, e.g. biocide dosing. In contrast, there were 5% of occasions when there was complete disagreement between qPCR and culture, and in all of these, qPCR would have indicated a requirement for emergency action that would require shutting down the system for an emergency disinfection and a careful review of the control programme. This small discrepancy, if anything, should enhance public health protection, as overall the results were comparable and the differences detected probably reflect the variation inherent in the techniques.

**Table 4:** Cooling towers – comparison of action and alert levels using quantitative PCR (qPCR) and culture for *Legionella pneumophila* and *Legionella* spp

		Culture No. (%)		
		
Target		Action (>10^4^ CFU l^−1)^	Alert (>10^3^ CFU l^−1)^	Satisfactory (<10^3^ CFU l^−1)^
*Leg. pneumophila*				
qPCR No. (%)	Action (>5 × 10^4^ GU l^−1^)	**7** (**3**)*	2 (1)	11 (5)
	Alert (>5 × 10^3^ GU l^−1^)	9 (4)	**10** (**4**)	9 (4)
	Satisfactory (<5 × 10^3^ GU l^−1^)	0 (0)	25 (11)	**159** (**69**)
*Legionella* spp.				
qPCR No. (%)	Action (>10^6^ GU l^−1^)	**5** (**2**)	9 (4)	41 (18)
	Alert (>10^5^ GU l^−1^)	11 (5)	**22** (**9**)	44 (19)
	Satisfactory (<10^5^ GU l^−1^)	3 (1)	8 (3)	**89** (**38**)

Figures in bold typeface represent those samples for which the results of both methods indicated the same action.

* The number and (%) of tests displaying the indicated result.

For cooling towers, the discrepancy between *Legionella* spp. qPCR in comparison to their detection by culture was much greater than that seen for *Leg. pneumophila*. In fact, *Leg. pneumophila* qPCR results corresponded more closely to the detection of *Legionella* spp. by culture than did *Legionella* spp. PCR (results not shown). This probably reflects the fact that, as mentioned above, the culture method was primarily developed for the detection of *Leg. pneumophila*. Indeed, *Leg. pneumophila* was the most common species detected in our samples, and this is generally true for all environmental samples using the standard method at 36°C.

The results for hot and cold water systems are summarized in Table 5. The numbers of *Leg. pneumophila* and *Legionella* species detected by qPCR could be adjusted to give reasonably comparable results to culture for *Leg. pneumophila* and Legionella spp., respectively. When using the *Leg. pneumophila* target, the proportion of exact matches (69%) was smaller than for cooling towers, but there were still only 4% of complete mismatches. Again, this was usually with higher qPCR suggesting immediate action when the culture result was satisfactory. Our results clearly showed that when the water temperature is high (>50°C), there is often a marked discrepancy between the qPCR and culture results for both *Leg. pneumophila* and *Legionella* species The systems chosen for monitoring in this study were selected because they were expected to have some legionellae in them because of their past history. The disparity of results seen at high temperatures is probably caused by the presence of legionellae in the water, in particular *Leg. pneumophila*, but being injured or killed by the higher temperatures and therefore not culturable. At lower temperatures, there was a much closer correlation between qPCR and culture for *Leg. pneumophila* in particular. In some cases, the disparities between results could be explained by the control measures in place affecting the cultivability of legionellae. For example, for Water E in Fig. 3, immediate control measures were taken following an initial high count of *Leg. pneumophila*. After this, the qPCR signal returned 3 weeks later and was about 10^3^ GU l^−1^ for 7/9 weeks although culture remained negative. This system was continuously dosed with chlorine dioxide which would have a similar effect to temperatures >50°C in injuring or killing the legionellae rendering them unculturable.

**Table 5:** Hot and cold water – comparison of action and alert levels using quantitative PCR (qPCR) and culture for *Legionella pneumophila* and *Legionella* species

		Culture number (%) with result		
		
Target		Action (>10^4^ CFU l^−1^)	Alert (>10^3^ CFU l^−1^)	Satisfactory (<10^3^ CFU l^−1^)
*Leg. pneumophila*				
qPCR No. (%)	Action (>4 × 10^4^ GU l^−1)^	**12** (**2**)	9 (2)	15 (3)
	Alert (>4 × 10^3^ GU l^−1)^	11 (2)	**30** (**6**)	79 (16)
	Satisfactory (<4 × 10^3^ GU l^−1)^	6 (1)	37 (7)	**306** (**61**)
*Legionella* spp.				
qPCR No. (%)	Action (>10^5^ GU l^−1)^	**10** (**2**)	12 (2)	42 (8)
	Alert (>10^4^ GU l^−1)^	14 (3)	**40** (**8**)	136 (27)
	Satisfactory (<10^4^ GU l^−1)^	5 (1)	33 (7)	**213** (**42**)

Figures in bold typeface represent those samples for which the results of both methods indicated the same action.

* The number and (%) of tests displaying the indicated result.

For cooling towers in particular, qPCR for *Leg. pneumophila* gave better correlation with culture than qPCR for *Legionella* species. However, *Leg. pneumophila* is clearly of the greatest public health significance, particularly in the nonhealthcare setting, so, in practice, monitoring cooling towers by qPCR for *Leg. pneumophila* will provide enhanced public health protection by enabling required actions to be taken in a much shorter time scale. If the results are considered in the context of repeat samples from a defined sample point or system, then overall qPCR produced a similar proportion of positive samples to culture. However, there were exceptions. In some cases, these were because of control measures being in place such as temperatures of >50°C and in others to the supplementary dosing of biocides in particular chlorine dioxide or chlorine. *Legionella pneumophila* detected by qPCR in these situations was almost certainly dead or injured. Detecting their presence, however, is of public health importance particularly in healthcare premises showing evidence of a continuing source of legionellae within the system. Indeed, in one hospital sampled throughout the study, *Leg. pneumophila* was detected frequently by qPCR but only rarely by culture. However, further nosocomial cases occurred after the trial had finished indicating that there were still viable and virulent *Leg. pneumophila* present in the system. This example illustrates the potential value of qPCR for indicating when an ongoing problem may not be adequately controlled. As for all microbiological monitoring, the results need to be reviewed in the context of the history of the system. Microbiological monitoring, in general, is of greatest value when it is used at a frequency that enables trend analysis.

**Figure 4:**
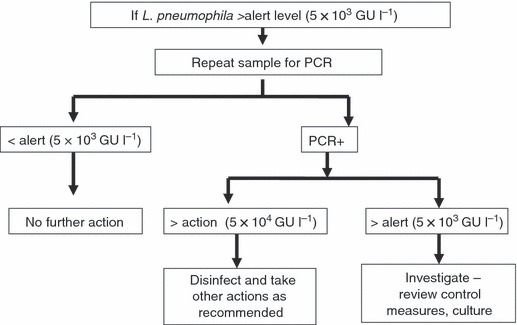
Suggested algorithm for interpretation of quantitative PCR results for routine monitoring cooling towers/water in nonhealthcare premises.

On the basis of this study and others, it is possible to derive algorithms for the use of qPCR for routine monitoring of cooling towers and water in nonhealthcare premises. A suggestion for cooling towers is shown in Fig. 4. This is based on *Leg. pneumophila* alone, as we have been unable to find evidence of other *Legionella* spp. causing cooling tower outbreaks. The sites chosen for sampling in this study were selected because they were known to have previously been colonized with legionellae and so were likely to yield some positive results. As a consequence, the NPVs recorded here were lower for hot and cold water samples than seen in other studies because of this site selection. Other studies of samples collected at random have shown the NPV of PCR is normally very high. So failure to detect legionellae by PCR is a strong indication that the system is safe. Similarly, at the action levels we have determined in this study, it would be reasonable to assume that if these are exceeded, the public health measures currently recommended within legislation and guidance when the culture action limit is exceeded should also be taken. We need to only consider how we deal with levels above the alert level. The alert level in Fig. 4 has been derived using the mean log difference of 0·71 establish in this study. In the algorithm given in Fig. 4, it is recommended that the sample is repeated, and if the subsequent result is less than the alert level, no further action is required. In the cases where the action level is exceeded, the system should be disinfected and other appropriate actions taken. If the alert level is exceeded, then they should be investigated, the control measures reviewed and a sample collected for culture. The culture will confirm if the PCR result is because of viable organisms and provide strains for further identification and typing if necessary.

**Figure 5:**
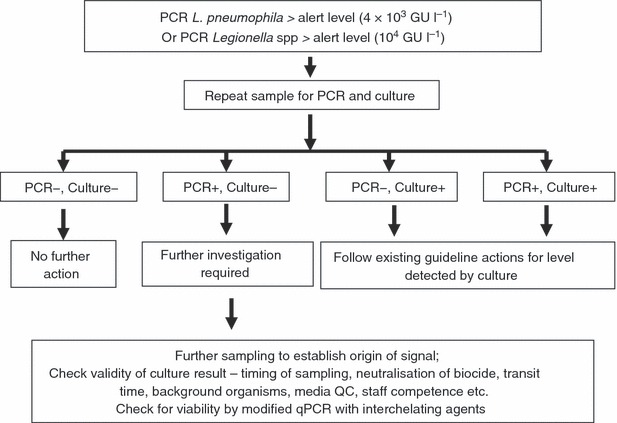
Suggested algorithm for interpretation of quantitative PCR results for water systems in healthcare settings and outbreak investigation.

A proposed algorithm for healthcare settings and outbreak investigations is shown in Fig. 5. PCR for both *Leg. pneumophila* and *Legionella* species is used in this instance because some other species are the cause of infections in these settings. Again, only results above the alert level but below the action level need consideration. We suggest the sample is repeated and tested for PCR and culture in parallel. Culture provides information on the viability of the signal and strains to analyse further if necessary for example for typing to compare with patient isolates. If both PCR and culture are positive or only the culture was positive, then the actions taken will be those recommended for monitoring by culture. In instances where PCR remains positive and culture negative, further investigation is required as this is clearly indicating a source of legionellae feeding into the system. This might require further sampling and review of the risk assessment to establish the origin of the signal and checks to ensure the validity of the culture method. Methods for distinguishing whether a PCR signal originates from living or dead cells are being developed (Nocker *et al.* 2006) and in the long term may largely overcome the need for culture except perhaps in outbreak investigation.

At present, the commercial charge for analysing water samples for *Legionella* by PCR is higher than the corresponding analysis by culture. Although for PCR the amount of labour per sample is appreciably less the cost of consumables and equipment is higher. However, with the increasing application of PCR, the costs of the reagent and equipment will inevitably decrease, and competition will inevitably drive down charges. Even at the current prices, the use of PCR can lead to appreciable savings for example by reducing the time that hospital wards or commercial plant may be required to be shut down from weeks to days.

In conclusion, culture remains the reference method currently; however, the lack of direct correlation between culture and PCR does not necessarily mean that culture is the more reliable or the most appropriate method for protecting the public health. Indeed in the future, culture may not necessarily be any longer considered to be the gold standard. We believe that qPCR action and alert levels can be adjusted to ensure public health is protected with the benefit that any remedial actions can be carried out in a much shorter time span which may prevent continued exposure to a system out of control for a period of several days. It will be important to ensure that any PCR method used has appropriate performance characteristics complying with agreed national and international standards and is at least as sensitive as the standard culture methods. When used to analyse equivalent volumes of water, the number of occasions when actions have to be taken is similar when using PCR as they would be when using culture. While there will probably be a small increase in the number of occasions when actions are taken as a result of qPCR results, these will be erring on the side of caution and therefore of potentially increased public health benefit.
